# HOXA1 Is an Antagonist of ERα in Breast Cancer

**DOI:** 10.3389/fonc.2021.609521

**Published:** 2021-08-18

**Authors:** Magali Belpaire, Bruno Ewbank, Arnaud Taminiau, Laure Bridoux, Noémie Deneyer, Damien Marchese, Gipsi Lima-Mendez, Jean-François Baurain, Dirk Geerts, René Rezsohazy

**Affiliations:** ^1^Louvain Institute of Biomolecular Science and Technology (LIBST), UCLouvain, Louvain-la-Neuve, Belgium; ^2^Pôle d’imagerie moléculaire, radiothérapie et oncologie (MIRO), Institut de Recherche Expérimentale et Clinique (IREC), UCLouvain, Woluwe-Saint-Lambert, Belgium; ^3^King Albert II Cancer Institute, Cliniques Universitaires St Luc, Woluwe-Saint-Lambert, Belgium; ^4^Department of Medical Biology, Amsterdam University Medical Centrum (AMC), University of Amsterdam, Amsterdam, Netherlands

**Keywords:** HOX proteins, estrogen receptor, NF-κB, endocrine therapy resistance, PBX

## Abstract

Breast cancer is a heterogeneous disease and the leading cause of female cancer mortality worldwide. About 70% of breast cancers express ERα. HOX proteins are master regulators of embryo development which have emerged as being important players in oncogenesis. HOXA1 is one of them. Here, we present bioinformatic analyses of genome-wide mRNA expression profiles available in large public datasets of human breast cancer samples. We reveal an extremely strong opposite correlation between *HOXA1 versus* ER expression and that of 2,486 genes, thereby supporting a functional antagonism between HOXA1 and ERα. We also demonstrate *in vitro* that HOXA1 can inhibit ERα activity. This inhibition is at least bimodal, requiring an intact HOXA1 DNA-binding homeodomain and involving the DNA-binding independent capacity of HOXA1 to activate NF-κB. We provide evidence that the HOXA1-PBX interaction known to be critical for the transcriptional activity of HOXA1 is not involved in the ERα inhibition. Finally, we reveal that HOXA1 and ERα can physically interact but that this interaction is not essential for the HOXA1-mediated inhibition of ERα. Like other HOX oncoproteins interacting with ERα, HOXA1 could be involved in endocrine therapy resistance.

## Introduction

Breast cancer is the most diagnosed female cancer and the leading cause of cancer mortality among women worldwide (1). The four commonly accepted molecular breast cancer subtypes are luminal A, luminal B, HER2-enriched, and basal-like tumors. Three main molecular markers are used to characterize these: ER (estrogen receptor), PR (progesterone receptor), and HER2 (human epithelial growth factor receptor) expression. Luminal A is defined as ER positive (ER+), PR positive (PR+), and HER2 negative (HER2−), and luminal B as ER+, PR+, and HER2-positive (HER2+) or HER2− (2, 3). ER+ cancers show the best prognosis since ER activity, which can drive oncogenesis, can be blocked using selective ER modulators (SERM) like Tamoxifen, aromatase inhibitors, or selective ER downregulators (SERD). However, many tumors develop resistance to such endocrine therapy, supporting the hypothesis that in addition to ERα expression, an alternative oncoprotein can become involved (4). HER2-enriched breast tumors are HER2+ and ER/PR−. Therapeutic antibodies targeting HER2 can inhibit tumor growth, but often resistance develops to this treatment. Finally, basal-like cancers do not express any of the three markers, ER, PR, and HER2, and are considered to be the most aggressive breast cancers. They are also characterized by cytokeratin 5 and 17 as well as EGFR expression. Basal-like breast cancers are sometimes also grouped as triple-negative breast cancers (TNBCs), but TNBCs do not share all basal-like characteristics (5).

ERα is expressed in about 70% of breast cancers (3). ERα is a transcription factor of the Nuclear Hormone Receptor (NHR) family. It is essential for mammary gland development, notably by mediating the mitogenic action of estrogens. Therefore, deregulation of its expression, abundance, stabilization, or degradation has considerable impacts on cell behavior and can trigger breast cancer development (6–8). ERα contains three well-characterized structural and functional domains: two transcription-activating regions and one DNA-binding domain. The first activating region, AF-1, is a hormone-independent transactivating domain. The second, AF-2, is the ligand-binding domain (LBD) and can regulate AF-1 activity (8).

*HOX* genes are master regulators of the embryo development. They code for a family of 39 evolutionary extremely well-conserved transcription factors that contain a homeodomain (9). *De novo HOX* gene expression has been reported in a wide variety of cancers, and several *HOX* genes can function as proper oncogenes (10–13). *HOXA1* was notably found to be involved in different types of cancer, including liver (14, 15), stomach (16), lung (17), prostate (18), endometrium (19), and breast cancers (20). *HOXA1 de novo* or overexpression appears systematically associated with cancer progression and poor prognosis. *HOXA1* is not expressed in the normal adult mammary gland but has been shown to be upregulated in some breast cancer tissues (20, 21). Experimental data show that *HOXA1* overexpression alone is sufficient to promote the oncogenic transformation of mammary epithelial cells (22). In *in vivo* models, Brock and colleagues confirmed the key role of *HOXA1* in mammary oncogenesis by demonstrating that silencing *HOXA1* with specific siRNAs decreases tumor incidence in mice (23).

At the molecular level, *HOXA1* expression can be activated by human growth hormone and E-cadherin. It stimulates cell survival and proliferation by activating p44/42 MAPK- and STAT-mediated gene transcription (22, 24–26). In contrast, *HOXA1* inhibition decreases expression of the anti-apoptotic protein BCL2 (20, 27).

We recently addressed the molecular mechanisms of HOXA1 action in breast cancer. We first provided evidence that PBX proteins, which are Three Amino acid Long Extension (TALE-) homeodomain proteins, are crucial HOXA1 cofactors during development (28). Next, we showed that disrupting the HOXA1-PBX interaction severely impairs HOXA1 oncogenic activity (29). This interaction depends on a hexapeptide motif shared by numerous HOX proteins. A WM-to-AA substitution in the hexapeptide indeed abrogated HOXA1’s ability to promote mammary cell proliferation, anchorage-independent cell growth, and contact inhibition loss, as well as its function in activating the expression of target oncogenes like the ephrin receptor gene *EphA2* (29–31).

Second, breast cancer transcriptome analysis highlighted a very strong positive correlation between HOXA1 and NF-κB pathway gene expression (32). These correlations were reinforced by the identification of several direct HOXA1 interactors that are important NF-κB pathway modulators (32, 33). At the functional level, Taminiau et al. revealed that HOXA1 can activate the NF-κB pathway and that this activation is important for cell proliferation and contact inhibition loss, in support of NF-κB activation as part of HOXA1 oncogenic activity. In addition, it was shown that NF-κB activation by HOXA1 occurs upstream of NF-κB nuclear translocation, probably at the level of signaling modulators like TRAF2 and RBCK1, *i.e.*, independent of HOXA1 transcription factor activity (32). NF-κB proteins are transcription factors involved in cellular processes like inflammation, apoptosis, and cell growth. Their deregulation can cause severe perturbation of cell physiology, and it has been widely accepted that NF-κB deregulation can trigger cancer (34, 35). Together, our previous reports clearly indicate that molecular mode of HOXA1 oncogenic activity is at least bi-modal since it requires the interaction with PBX or with NF-κB modulators.

In this study, we present additional breast cancer transcriptome analysis identifying an extremely strong, inverse correlation between genes associated with *HOXA1* expression and with ER status: genes that are upregulated in the presence of *HOXA1* expression appear downregulated upon ERα activation, and *vice versa*. These opposite correlations led to explore the hypothesis that HOXA1 and ERα could display antagonistic activities.

## Materials and Methods

### Bioinformatic Analysis of Public Genome-Wide Breast Cancer mRNA Expression Datasets

Genome-wide mRNA expression profiling datasets of human breast cancer samples in the public domain (n=45) were retrieved from the NCBI Gene Expression Omnibus (GEO^1^), EMBL European Bio-informatics Institute (EMBL-EBI^2^), or NIH-TCGA^3^ websites on December 31, 2018. Datasets with <100 tumor samples (n=19) were excluded. Of 26 unique datasets remaining, 14 were excluded because of incomplete clinical annotation or because only specific breast cancer subtypes were included (n=11 and n=3, respectively, for details see **Supplemental Table 1**). These final 12 datasets were analyzed using R2: a genomic analysis and visualization platform^4^ developed in the Department of Oncogenomics at the Amsterdam University Medical Centre–University of Amsterdam, Netherlands, as described in (36). In addition, for **Figure 2**, the Bergh-159 dataset (GSE1456) was analyzed. The TranscriptView genomic analysis and visualization tool within R2 was used to check if probe-sets uniquely mapped in an antisense orientation to an exon of their target gene^5^. All probe-sets in this study meet these criteria. All expression values and other details of the datasets can be obtained through their GSE, E, or TCGA number from the NCBI-GEO, EMBL-EBI, and NIH-TCGA websites, respectively.

The results of the correlation between *ESR1* and *HOXA1* respective expression profiles and the rest of the breast cancer transcriptomes were split in two lists named *HOXA1+/ESR1−* and *HOXA1−/ESR1+*, which hold, respectively, the genes whose expression profiles correlated positively with the expression of *HOXA1* and negatively with the expression of *ESR1* and the list of those that correlated negatively with *HOXA1* and positively with *ESR1*. We performed functional enrichment analyses on those lists using the http://geneontology.org/ interface, using Fisher’s exact test and corrected for multiple testing following the FDR method. The reference functions were Gene Ontology (GO) molecular function, GO biological process, and GO cellular component.

To assess the relation between *HOXA1* and *ESR1* expression values and survival, we generated Kaplan–Meier plots using the R2 interface. We used the Kaplan Scan feature to split the samples according to the level of expression of *HOXA1* and *ESR1*, respectively, resulting in one split of samples assigned to either high or low *HOXA1* expression and a second split corresponding to either high or low *ESR1* expression. The Kaplan Scan feature establishes the optimum survival cut-off based on a logrank test as described in (37). To reveal the effect of the combination of the expression of *ESR1* and *HOXA1* in survival probability, we performed Kaplan-Meier analysis combining the groups resulted from the Kaplan scan into the four possible combinations of high and low values of expression of *HOXA1* and *ESR1* expression (R2 Kaplan by combination of two categorical tracks).

### Plasmid Constructs

Reporter plasmids as well as PREP1, PBX1A, HOXA1, and IκB super repressor (IκB-SR) expression vectors have been previously described (**Table 1**).

**Table 1:** Previously described expression vectors and reporter plasmids.

Plasmids	References
pML-EPHA2-r4-Luc (short name: *EphA2::luc*)	(30)
pGL4.32[luc2P/NF-κB-RE/Hygro] (short name: *NF-κBluc*)	(Promega)
pCMV-LacZ (short name: *CMV::lacZ*)	(38)
pCS2-PREP1	(39)
pCMV-PBX1A	(40)
IκB super repressor (short name IκB-SR)	(41)
pEXP-Flag(Nter)-mHOXA1	(33)
pEXP-GST(Nter)-mHOXA1	
pEXP-VC155(Nter)-mHOXA1	
pEXP-Flag(Nter)-mHOXA1^Δ71-199^	(42)
pEXP-Flag(Nter)-mHOXA1^ΔHD^	
pEXP-Flag(Nter)-mHOXA1^WM-AA^	
pEXP-Flag(Nter)-mHOXA1^WFQN-SVAA^	
pEXP-GST(Nter)-mHOXA1^Δ71-199^	
pEXP-GST(Nter)-mHOXA1^ΔHD^	
pEXP-GST(Nter)-mHOXA1^WM-AA^	
pEXP-GST(Nter)-mHOXA1^WFQN-SVAA^	
pEXP-VC155(Nter)-mHOXA1^Δ71-199^	
pEXP-VC155(Nter)-mHOXA1^ΔHD^	
pEXP-VC155(Nter)-mHOXA1^WM-AA^	
pEXP-VC155(Nter)-mHOXA1^WFQN-SVAA^	

The pSG5-hERα expression vector and the 3xERE::luc-TATA reporter vector (hereafter called *ERE::luc*), which includes the estrogen response element of the vitellogenin A2 gene promoter, were kind gifts from Han Weidong (Chinese PLA General Hospital, Beijing, China).

pENTR-ESR1 was obtained from the ORFeome v5.1^6^, thanks to Jean-Claude Twizere (Molecular Biology of Diseases, GIGA, ULiège, Belgium). The expression vectors pEXP-Flag(Nter)-ESR1, pEXP-GST(Nter)-ESR1, and pEXP-VN173(Nter)-ESR1 were generated by Gateway^®^ technology (Invitrogen, Carlsbad, USA), with an LR clonase^®^ reaction between pENTR-ESR1 and pDEST-Flag(Nter), pDEST-GST(Nter), or pDEST-VN173(Nter), respectively. Similarly, pENTR-ESR1^AB^, pENTR-ESR1^CDEF^, and pENTR-ESR1^EF^ were generated by BP clonase^®^ reaction between pDONR223 and PCR products obtained from pGEX2TK-ESR1^AB^, pGEX2TK-ESR1^CDEF^, and pGEX2TK-ESR1^EF^ (43), respectively. These three plasmids were kind gifts from Sylvie Mader (Molecular Targeting in Breast Cancer Treatment Research Unit, Université de Montréal, Montréal, Canada). The attB-flanked PCR products were obtained with the following primers (**Table 2**): (1) and (2) for *ESR1^AB^*; (3) and (4) for *ESR1^CDEF^*; (5) and (6) for *ESR1^EF^*. LR clonase^®^ reactions were then performed with pDEST-VN173(Nter) to obtain the three corresponding pEXP vectors.

**Table 2:** Primers used to generate deletion derivatives of the *ESR1* gene.

Primer #	Sequences
(1)	GGGGACAACTTTGTACAAAAAAGTTGGCaccctccacaccaaagcatctgg
(2)	GGGGACAACTTTGTACAAGAAAGTTGGGTAgtagcgagtctccttggcagattcc
(3)	GGGGACAACTTTGTACAAAAAAGTTGGCctgtgcagtgtgcaatgactatgc
(4)	GGGGACAACTTTGTACAAGAAAGTTGGGTAtcagaccgtggcagggaaaccc
(5)	GGGGACAACTTTGTACAAAAAAGTTGGCccagagagatgatggggagggc
(6)	GGGGACAACTTTGTACAAGAAAGTTGGGTAtcagaccgtggcagggaaaccc

Similarly, pEXP-Flag(Nter)-hHOXA1 and pEXP-VC155(Nter)-hHOXA1 were generated from the pENTR-hHOXA1 from the ORFeome and from the pDEST-Flag(Nter) and pDEST-VC155(Nter), thanks to the Gateway^®^ technology (Invitrogen).

### Cell Culture and Transfection

The MCF10A, MCF7, HEK293T, and COS7 cell lines were maintained and transfected as described in (32).

### Western Blotting

Seven hundred thousand HEK293T cells were seeded per well of six-well plates and transfected with combinations of plasmids encoding Flag- or GST-tagged proteins. Twenty-four hours after transfection, cells were rinsed once with PBS and then lysed in cold IPLS buffer (20 mM Tris-HCl pH7.5, 120 mM NaCl, 0.5 mM EDTA, 0.5% NP40, 10% glycerol) supplemented with 1× cOmplete™ protease inhibitor cocktail (#11697498001, Merck, Darmstadt, Germany), during 20 min on ice under gentle agitation. Cell lysates were centrifuged 5 min at 16,000 g and 4°C, sonicated for 15 s, and then boiled at 95°C for 5 min. Expression of Flag- or GST-fused proteins and ACTIN was analyzed by Western-blotting with primary mouse anti-Flag (#F1804, Merck), or anti-GST antibody (#G1160, Merck) or anti-ACTIN antibody (#A3854, Merck), respectively, and HRP-coupled secondary anti-mouse IgG (#sc-516102, Santa Cruz Biotechnology). Primary Flag- or GST-antibodies were diluted 1:5,000 in 10% milk in TTBS, anti-ACTIN was diluted 1/20,000 in TTBS, and the secondary anti-mouse IgG antibody was diluted 1/10,000 in 1% milk in TTBS. The ACTIN signal was used as a protein loading control.

### Glutathione Co-Precipitation

Seven hundred thousand HEK293T cells were seeded per well of a six-well plate and transfected with combinations of plasmids encoding Flag- or GST-tagged proteins. Empty pDEST-GST(Nter) vector was used as a negative control. Proteins were harvested 48 h post-transfection as described above, but without sonication. Thirty µl of glutathione-sepharose beads (#GE17-0756-01, Sigma-Aldrich, St. Louis, USA) were washed three times with cold IPLS and then added to protein lysates overnight at 4°C on a rotating wheel. Beads were then washed three times with cold IPLS. The first wash was stored to assess the abundance of unbound GST-fusion protein. The beads were then resuspended in Laemmli loading buffer and boiled for 5 min at 95°C. Expression of Flag- or GST-fused proteins was detected as described above. Detection of the Flag epitope reveals the presence or absence of an interaction between the two proteins tested. Detection of the GST tag allows evaluating the abundance of bead-bound GST-protein.

### Bimolecular Fluorescence Complementation

Seventy-five thousand COS7 cells were seeded on glass coverslips in 24-well plates and were transfected 16 h growth later with 250 ng of pEXP-VC155(Nter) and 250 ng of pEXP-VN173(Nter) plasmids encoding HOXA1 and ERα fusion proteins, respectively. Empty pDEST-VC155(Nter) and pDEST-VN173(Nter) vectors were used as negative controls. Twenty-four hours post-transfection, cells were washed twice with PBS and fixed for 20 min with 4% PFA-PBS (#441244, Sigma-Aldrich) at room temperature. Cells were then rinsed twice for 5 min in TBS-T buffer (50 mM Tris-HCl, pH 7.5, 155 mM NaCl, 0.1% Triton X-100 (#10789704001, Merck)) and once for 10 min with TB buffer (50 mM Tris-HCl, pH 7.5). Cells on coverslips were stained in a mounting medium containing DAPI and Vectashield (#H-1200, Labconsult, Brussels, Belgium), and pictures were taken under an epifluorescence microscope (Axioskop 2, Zeiss, Oberkochen, Germany). Fluorescence was quantified with the IMAGEJ software and tested interactions were considered as positive when the emitted fluorescence was at least three times higher than in the negative control conditions. pEXP-VN173(Nter)-hHOXA1 with pEXP-VC155(Nter)-mHOXA1 was used as a positive BiFC control in each experiment.

### Reporter Assays

Two hundred thousand MCF10A cells per well were plated on 24-well plates and transfected with the following plasmids: 250 ng of luciferase reporter plasmid (*ERE::luc* or *EphA2::luc* or *NF-κB::luc*), 50 ng of *CMV::lacZ*, 250 ng of pEXP-Flag(Nter)-hHOXA1, and/or 100 ng of pCS2-Prep1 and 100 ng of pCMV-Pbx1a, and/or 250 ng of pSG5-hERα and/or 250 ng of IκB-SR expression vectors, for a total of 1 µg of DNA per well. For assays involving mHOXA1 deletion variants, 250 ng of *ERE::luc* and 50 ng of *CMV::LacZ* reporter plasmids were transfected together with 250 ng of pSG5-hERα and/or 250 ng of pEXP-Flag(Nter)-mHOXA1, -mHOXA1^ΔHD^, ^ΔCenter^, ^WM-AA^, or ^WFQN-SVAA^ expression vectors. The total amount of DNA was kept equal for all conditions by the addition of carrier pCAT vector when required. Each condition was tested in duplicate, and each experiment was carried out at least three times. Twenty-six hours post-transfection, cells were harvested. Luciferase and β-galactosidase activities were measured with a high-sensitivity Luciferase (#11669893001, Roche, Penzberg, Germany) and a chemiluminescent β-galactosidase assay (#11758241001, Roche), respectively, following the manufacturer’s instructions. Luciferase activity was normalized using constitutive β-galactosidase activity. Experiments with HOXA1 variants were conducted using the Dual-Light™ Luciferase & β-Galactosidase Reporter Gene Assay System (#T1003, Thermo Fisher Scientific, Waltham, USA) according to the manufacturer’s instructions, except that cell extracts were obtained by collecting cells in 100 µl lysis buffer.

### Statistical Analysis

**Figure 1:**
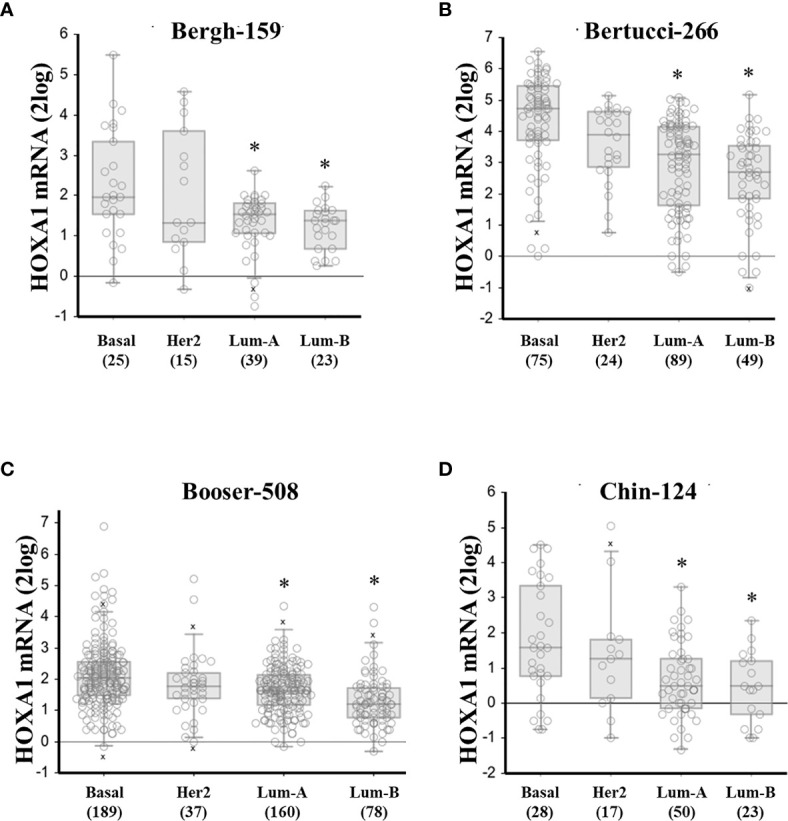
High *HOXA1* mRNA expression correlates with the basal breast cancer subtype. *HOXA1* mRNA expression correlation with breast cancer molecular subtypes. Panels **(A–D)** represent the results from all 4 breast cancer datasets in the public domain with sample number > 100, and annotation on molecular subtype: Bergh-159, Bertucci-266, Booser-508, and Chin-124, respectively. Below the graphs are the different subtypes: basal-like (basal), HER2-overexpressing (Her2), Luminal-A (Lum-A), and Luminal-B (Lum-B), between brackets are the number of samples per subtype. mRNA expression values for the individual samples are presented as open circles, horizontal bars represent (from up to down: maximum value, [upper quartile, median value, lower quartile – boxed], and minimal value. Outlier samples (more or less than 3/2 of upper or lower quartile, respectively) are denoted by “x”. * denotes significant difference with the basal subtype expression (p < 0.05, Welch’s ANOVA with post-hoc test, significant differences found for both normal and 2log-transformed expression values).

**Figure 2:**
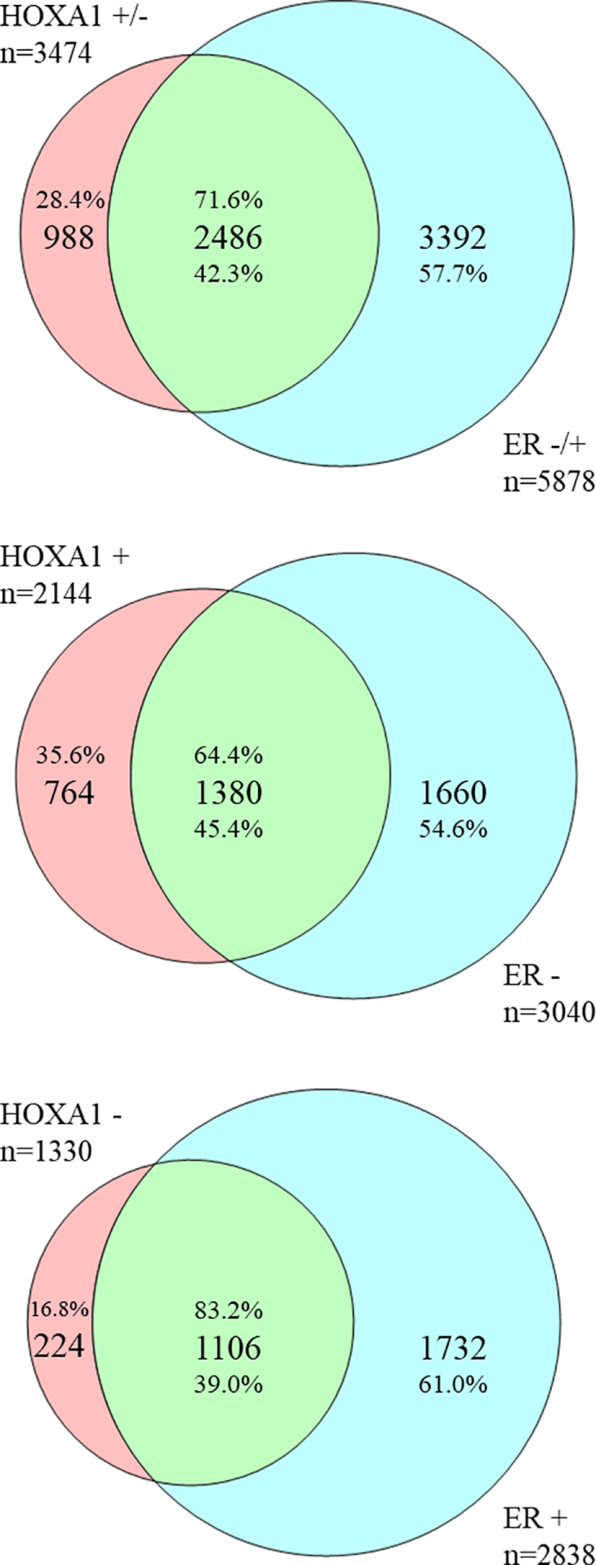
HOXA1- and ER status-correlating genes in breast cancer correlate inversely. Overlap between HOXA1- and ER status-correlating genes extracted from public genome-wide mRNA expression breast cancer datasets. Venn diagrams represent the large overlap between genes whose mRNA expression correlates with that of *HOXA1*, and with ER status. Top diagram: Of 3474 genes correlated with *HOXA1* expression, the majority, 2486 genes (71.6%), show an inverse correlation with ER status. This is 42.3% of genes correlated with ER status (5878 genes total). Middle diagram: Of 2144 genes positively correlated with HOXA1 expression, a smaller majority, 1380 genes (64.4%), show an inverse correlation with negative ER status. This is 45.4% of genes oppositely correlated with ER status (3040 genes total). Bottom diagram: Of 1330 genes correlated with *HOXA1* expression, a large majority, 1106 genes (83.2%), show an inverse correlation with positive ER status. This is 39.0% of genes correlated with positive ER status (2838 genes total). The results are in support of an opposite correlation between *HOXA1* mRNA expression and ER status in breast cancer, and suggest that especially genes negatively associated with *HOXA1* mRNA, e.g. genes potentially downregulated by HOXA1, are involved in ER status in breast cancer.

For the bio-informatic analyses in **Figures 1**, **2**, **Table 3**, and **Supplemental Tables 1**–**3**, *HOXA1* mRNA expression was correlated to mRNA expression of other genes using a 2 log Pearson test. The significance of a correlation is determined by t = R/sqrt((1-r2/(n-2)) where R is the correlation value and n is the number of samples. Distribution measure is approximately as t with n-2 degrees of freedom (see ^7^ for details). *HOXA1* mRNA expression correlations with breast cancer clinical parameters in **Table 3** and **Figure 1** were determined using the non-parametric Kruskal-Wallis test. For all tests, differences were considered significant if p < 0.05.

**Table 3:** *HOXA1* mRNA expression in breast cancer datasets.

Dataset	ER status			HER2 status	PR status		Correlating genes		Array		Deposition	
Name - Size	Correlation	P value	ER-/ER+	P value	Correlation	P value	HOXA1	ER status	Platform	Norm.	GSE/EBI	PubMed
Bertucci - 266	Negative	1.19 10^-12^	113/150	n.s.	Negative	2.69 10^-9^	7768	9578	Affy U133P2	MAS5.0	21653	20490655
Booser - 508	Negative	3.05 10^-10^	205/297	n.s.	Negative	4.40 10^-5^	7366	6308	Affy U133A	MAS5.0	25066	21558518
Chin - 124	Negative	0,021	44/80	n.s.	Negative	0,028	2558	5031	Affy U133A	MAS5.0	E-TABM-158	17157792
Clynes - 104 (121)	Negative	0,015	34/67	n.d.	n.d.	n.d.	2685	3971	Affy U133P2	MAS5.0	42568	23740839
EXPO Breast - 351	Negative	0,044	75/150	n.s.	n.s.	n.s.	5204	8808	Affy U133P2	MAS5.0	2109	not yet
Halfwerk - 947	Negative	2.03 10^-8^	200/581	n.s.	Negative	7.15 10^-5^	9325	7246	Affy U133A	Complex	several*	several**
Iglehart - 123	Negative	0,038	50/73	n.s.	n.d.	n.d.	2931	9196	Affy U133P2	MAS5.0	5460	18297396
Miller - 251	Negative	6.78 10^-3^	34/213	n.d.	Negative	0,030	4047	4635	Affy U133A	MAS5.0	3494	16141321
Prat - 156	n.s.	n.s.	52/104	n.s.	Negative	1.91 10^-3^	1155	2184	Affy U133P2	MAS5.0	50948	24443618
TCGA - 1097	Negative	6.18 10^-25^	238/808	n.s.	Negative	4.59 10^-12^	14524	15161	tcgars	rsem	BRCA-1098	not yet
TCGA - 528	Negative	2.55 10^-4^	117/404	n.s.	n.s.	n.s.	7741	12143	AgilentG4502	custom	BRCA-593	23000897
Wang - 286	Negative	8.73 10^-3^	77/209	n.d.	n.d.	n.d.	4602	5708	Affy U133A	MAS5.0	2034	15721472

HOXA1 mRNA expression was investigated for correlation to clinical parameters in public genome-wide mRNA expression breast cancer datasets. Data were downloaded and analysed as described in the Materials and Methods. The first column represents name and size of the dataset. Columns 2-4 represent ER tumour status; sign and p value of correlation with HOXA1 mRNA expression, sample numbers with ER- versus ER+ status, respectively. Column 5 represents the correlation between HOXA1 mRNA expression and HER2 tumour status. Columns 6 and 7 represent sign and p value of the correlation between HOXA1 mRNA expression and PR tumour status, respectively. Columns 8 and 9 represent the amount of genes whose expression correlates with HOXA1 mRNA expression and ER tumour status, respectively. Columns 10 and 11 represent array type and data normalization method, respectively. Columns 12 and 13 represent data set deposition: NCBI-GEO/EMBL-EBI dataset identifier and PubMed ID, respectively. * Dataset is compiled of GSE1456/3494/5327/6532/7390 and EBI E-TABM-158. ** PubMed ID: 16813654, 29136509, 17420468, 20479250, 25788628, 17157792, respectively. n.d. means no data, n.s. means test result not significant (p > 0.05).

The *in vitro* data were analyzed using mixed models in R (lmer function of the lmerTest R package) (44) and SAS 9.4. (mixed procedure). A log-transformation is applied on the response variable to meet the mixed model assumptions (normality and homogeneity of the residuals). Significant differences between groups were analyzed using *post-hoc* comparison tests with Bonferroni correction to ensure the level alpha (= 0.05) in a multiple comparison test setting (45) or through a Tukey test.

## Results

### Bioinformatic Analysis of Public Genome-Wide Breast Cancer mRNA Expression Datasets

We previously identified a causal role between HOXA1 expression and NF-κB pathway activation in breast cancer (32). To further explore an oncogenic role for HOXA1 in breast cancer, we examined *HOXA1* mRNA expression in public genome-wide mRNA expression datasets of human breast cancer samples (**Table 3**). We analyzed whether *HOXA1* expression levels were correlated to the three main breast cancer molecular markers: ER, PR, and HER2. *HOXA1* mRNA expression showed significant inverse correlation to ER status and PR status of breast cancer samples, in 11 of 12 datasets. Also, PR status shows this opposite correlation with *HOXA1*, in seven of nine datasets with PR status annotation. *HOXA1* expression was not significantly correlated to HER2 tumor status in any of the datasets. These results strongly suggest that ERα and PR tumor expression, and thereby the potential for ERα or PR pathway activation, is decreased in the presence of HOXA1.

Interestingly, *HOXA1* expression was significantly correlated to breast cancer molecular subtypes as well. An analysis of the four public datasets annotated for (PAM50) molecular subtypes showed that *HOXA1* was consistently most highly expressed in basal-like samples, compared to HER2 or luminal subtypes, confirming the results in **Table 3**, and in further support of a role for HOXA1 in breast cancer aggressiveness (**Figure 1** and **Supplemental Figure 1**).

Since especially the correlation between *HOXA1* mRNA expression and (negative) ER status was significant, in 11 of 12 datasets tested, suggesting that HOXA1 could repress ERα expression, or vice versa, we wanted to further define the possible signaling pathways involved. To this end, we downloaded all genes whose mRNA expression correlated with *HOXA1* mRNA expression or with tumor ER status (see **Table 3**). To obtain biologically and statistically robust results, we only included genes that showed significant expression correlation in at least 6 of 12 datasets analyzed, with the extra criterion that the correlations needed to carry the same sign: be positive (e.g., high gene mRNA expression correlates with high *HOXA1* mRNA expression) or negative (e.g., low gene mRNA expression correlates with high *HOXA1* mRNA expression), with a penalty for conflicting correlations (see *Materials and Methods*). We found 5,878 genes with significant, sign-consistent correlations to ER status in at least six of 12 datasets, and 3,474 genes that correlated to *HOXA1* mRNA expression using the same criteria (**Supplemental Table 2**). Comparison of the two gene lists showed a very large overlap: 2,555 genes were significantly correlated to both *HOXA1* mRNA expression and ER status (**Supplemental Table 3**). Importantly, 2,486 of 2,555 genes (97.30%) showed inverse correlation: opposite correlation to *HOXA1* mRNA expression, but positive correlation to ER status (1,106 genes; 43.29%) or positive correlation to *HOXA1* mRNA expression, but opposite correlation to ER (1,380 genes; 54.01%). Overlap analysis also showed that especially genes oppositely correlated to *HOXA1* mRNA expression but positively to ER expression are enriched, suggesting that HOXA1 could be involved in the downregulation of these genes and thereby act as an ERα repressor in breast cancer (**Figure 2**). Enrichment analysis of these distinct gene sets showing inverse correlation with HOXA1 *versus* ER expression according to Gene Ontology (GO) supports that when *HOXA1* is upregulated while the ER gene *ESR1* is downregulated, tumors show enhanced cytokine and chemokine signaling, as well as enhanced immune response (**Supplemental Table 4**).

To determine to what extent the HOXA1 *versus* ER expression status might be clinically relevant, we assessed the effect of HOXA1 and ER expression on the relapse-free or overall survival probability of patients, and we generated Kaplan-Meier plots with the R2 genomics analysis platform. Out of the 12 datasets used, only four had survival information (**Supplemental Figure 2**). For three datasets, the combined expression status for *HOXA1* and *ESR1* revealed distinct outcomes supportive of a functional interaction between HOXA1 and ER. Low *HOXA1* associated with high *ESR1* expression is significantly associated to the best survival probability, while high *HOXA1*-low *ESR1* appears as the worst or the second worst condition. Comparing survival curves corresponding to high *ESR1*, expression of *HOXA1* (high or low HOXA1) clearly shows an impact. Reciprocally, comparing survival curves corresponding to high *HOXA1*, expression of *ESR1* (high or low *ESR1*) also shows an effect. This supports a functional interaction between HOXA1 and ER in breast cancer, *ESR1* expression improving the *HOXA1*+ condition, while *HOXA1* expression worsening the survival probability of ER+ patients.

### HOXA1 Inhibits ERα Activity Independently of the Cofactors PREP and PBX

**Figure 3:**
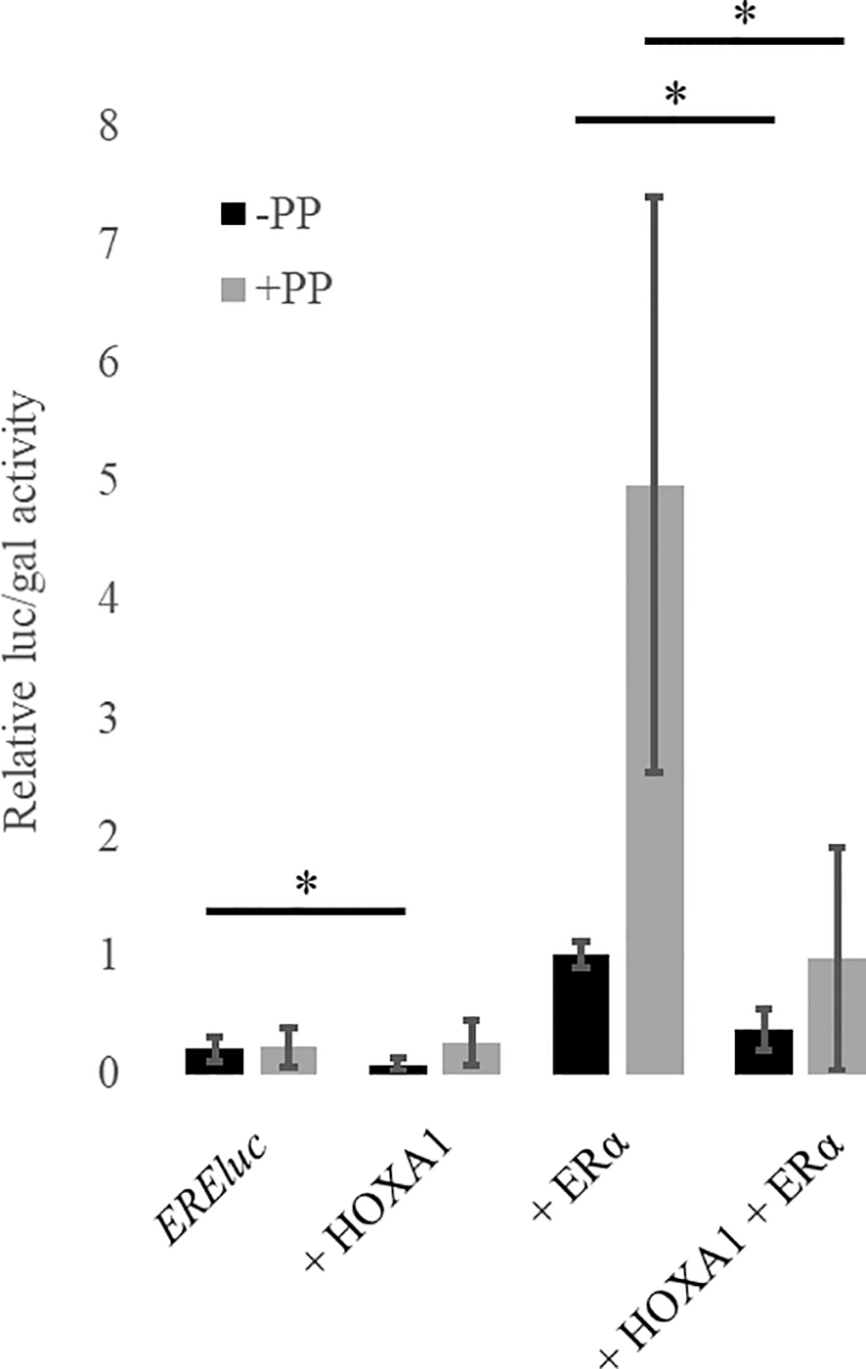
HOXA1 inhibits ERα activity in presence and in absence of the cofactors, PREP1 and PBX1A. MCF10A cells were transfected with plasmids encoding ERα, Flag-HOXA1 along with *CMV::lacZ* and *EREluc* without (black boxes) or with (grey boxes) PREP1 and PBX1A (PP). ER-mediated luciferase activity (luc) was reported to the β-galactosidase activity (gal) (relative luc/gal activity). The activation of the ER reporter by ERα is reduced in presence of HOXA1 (black) and this effect is also observed in the presence of PREP1 and PBX1A (grey) (N≥3). The relative *EREluc* activity in presence of ERα was set to 1. * means p < 0.05 (N≥3, n=2). Error bars represent standard deviation; for N experiments and n replicates per experiment.

To assess the HOXA1-ERα functional antagonism suggested by the bio-informatic analyses above, HOXA1 and ERα activities and their possible interactions were analyzed *in vitro*. HOXA1 and ERα target gene reporter assays were carried out to establish whether HOXA1 might interfere with ERα activity, and *vice versa*. Human mammary epithelial cells MCF10A were transfected with plasmids encoding ERα, HOXA1, PREP1, and PBX1A and the *ERE::luc* ER activity reporter. This *ERE::luc* reporter contains the gene encoding the luciferase under the control of three ER-binding core sequences from the vitellogenin A2 gene promoter. As expected, ERα is able to activate *ERE::luc.* HOXA1 alone does not activate *ERE::luc*, but rather decreases the activity of the reporter (**Figure 3**). In addition, in the presence of HOXA1, *ERE::luc* activation by ERα significantly decreased, demonstrating that HOXA1 can inhibit the transcriptional activity of ERα.

HOXA1 transcriptional activity critically relies on its interaction with PBX proteins (28, 40, 46). Magnani *et al.* uncovered that PBX1 and ERα share a large proportion of their respective target gene promoter-binding sites in MCF7 human mammary cancer cells, and suggested that ERα and PBX could physically interact (47). Since PBX1 is a shared partner between HOXA1 and ERα, we considered that the HOXA1-ERα antagonism could act through competition for PBX1. We therefore tested the effect of PBX1A on HOXA1 and ERα activity. To promote PBX nuclear entry, we also included PREP1 in the assay (48). MCF10A immortalized normal human mammary cells were transfected as described above, with the addition of plasmids encoding PREP1 and PBX1A. Addition of PREP1 and PBX1A significantly increased ERα activation of the *ERE::luc* reporter. However, HOXA1 still inhibited ERα activity, showing that HOXA1 can interfere with the ERα ability to stimulate transcription, both in the presence or the absence of PREP1 and PBX1A (**Figure 3**).

### HOXA1 DNA Contact Is Important for ERα Inhibition

**Figure 4:**
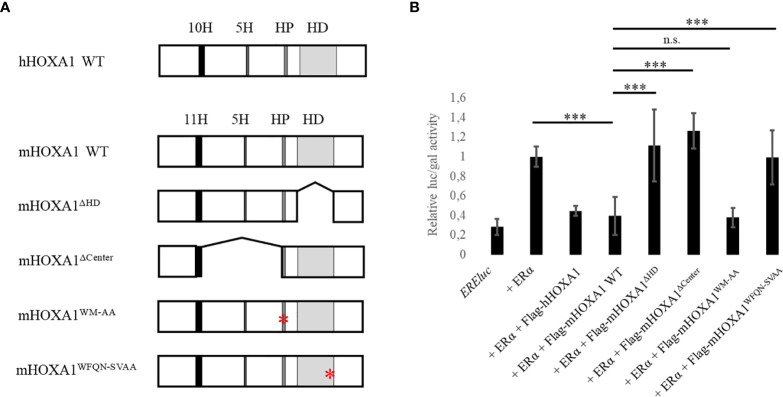
Map of HOXA1 variants and their impact on ERα activity. **(A)** hHOXA1 WT (336 amino acids) encompasses two histidines stretches of ten (10H) and five amino acids (5H), a hexapeptide (HP) and a homeodomain (HD) of sixty amino acids structured in three α-helices. mHOXA1 WT (331 amino acids) shares 94,7% identity with hHOXA1 WT. A major difference is the length of the first histidine tract showing eleven amino acids in the mouse. mHOXA1^ΔHD is^ deprived of the homeodomain, responsible for the loss of DNA binding. mHOXA1^ΔCenter^ lacks the 129-amino acid sequence between the first histidine repeat and the hexapeptide. mHOXA1^WM-AA^ shows a mutation in the sequence of the hexapeptide, perfectly conserved between the human and mouse proteins and changed from TFDWMK to TFDAAK, which implies the loss of PBX interaction. mHOXA1^WFQN-SVAA^ harbours a mutation in the third α-helix of the homeodomain, which impairs DNA binding. **(B)** Impact of HOXA1-derivatives on the ERα transcriptional activity. MCF10A cells were transfected with plasmids encoding EREluc, ERα, Flag-hHOXA1, Flag-mHOXA1 WT, Flag-mHOXA1^ΔHD^, Flag-mHOXA1^ΔCenter^, Flag-mHOXA1^WM-AA^, Flag-mHOXA1^WFQN-SVAA^ and β-galactosidase under the control of a CMV promoter. ER-mediated luciferase activity (luc) was reported to the β-galactosidase activity (gal). Activation of the ER reporter by ERα is reduced in the presence of Flag-hHOXA1 and Flag-mHOXA1 WT. This effect is lost with the deletion or the mutation of the homeodomain, as well as upon the deletion of the central part of the protein. Mutations in the hexapeptide does not affect the ability of HOXA1 to inhibit ERα (N=3, n=2). n.s. means test result not significant (p > 0.05), *** means p < 0,001. Error bars represent standard deviation, for N experiments and n replicates per experiment.

To characterize the functional interaction between HOXA1 and ERα, we performed luciferase reporter assays with murine HOXA1 (mHOXA1) mutant variants (mHOXA1 shares 94.7% sequence identity with human HOXA1, hHOXA1). HOXA1 protein contains two histidine repeats, a so-called hexapeptide motif shared by most HOX proteins and a homeodomain, as depicted in **Figure 4A**. The hexapeptide is a six amino acid hydrophobic sequence involved in PBX interaction. The homeodomain is the only DNA-binding domain of HOX proteins (9), but it also contributes to protein-protein interactions (42, 49–51).

The mHOXA1 mutant variants assayed are the following. mHOXA1^ΔHD^ lacks the homeodomain, and mHOXA1^WFQN-SVAA^ displays four amino acid substitutions in the third helix of the homeodomain. These two HOXA1 variants are impaired in their DNA-binding capacity. mHOXA1^Δ71-199^ (hereafter referred to as mHOXA1^ΔCenter^) lacks a central region of the protein, extending from amino-acid 71 to 199. mHOXA1^WM-AA^ has a mutant hexapeptide and has lost its capacity to interact with PBX (32, 46, 52, 53). MCF10A cells were transfected with HOXA1 expression plasmids to assess their effect on ERα activity, as determined by *ERE::luc* activity. Like the hHOXA1 protein, mHOXA1 was efficient in inhibiting ERα. mHOXA1 variants with decreased DNA binding did not impair ERα activity, but mHOXA1^WM-AA^ still could (**Figure 4B**). This result corroborates the observation that the HOXA1-mediated ERα inhibition does not rely on PBX, i.e., the ability of HOXA1 to interact with PBX. Next, these results also suggest that the capacity of HOXA1 to bind DNA through its homeodomain is important for the ERα inhibition.

### HOXA1 and ERα Can Interact in the Cell Nucleus

**Figure 5:**
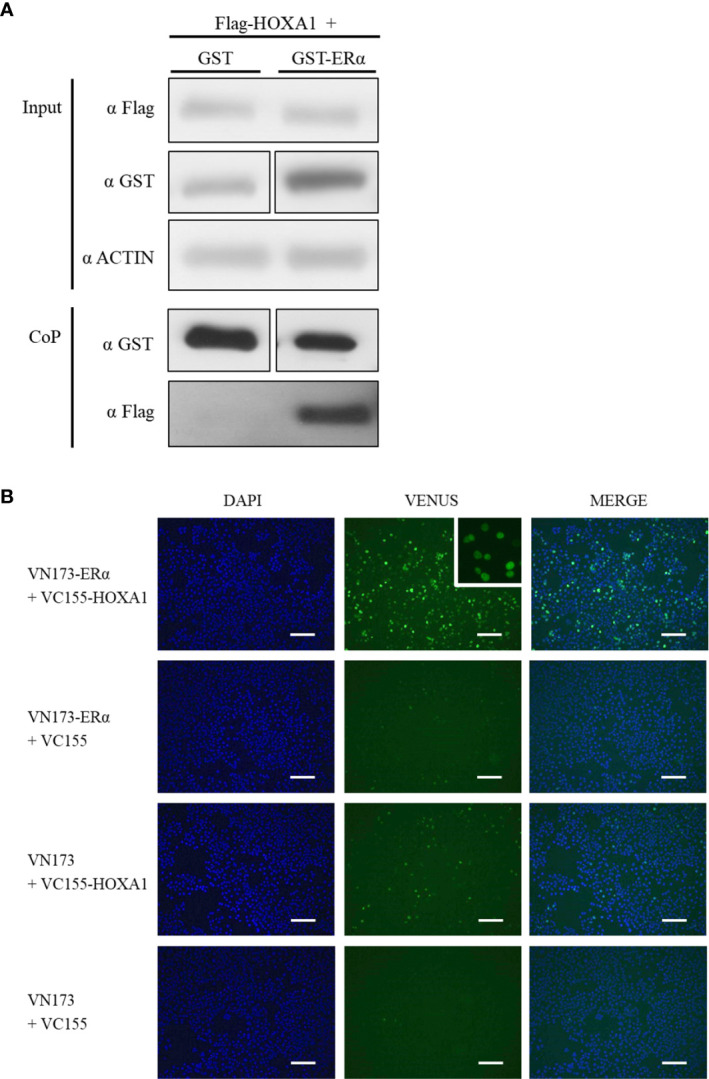
Interaction between human HOXA1 and ERα in co-precipitation and BiFC assays. **(A)** For co-precipitation assays, HEK293T cells were transfected with plasmids encoding Flag-HOXA1 and GST or GST-ERα. Protein abundance was monitored prior to co-precipitation with anti-Flag and anti-GST antibodies (Input). β-actin was used as a loading control. After co-precipitation (CoP), Flag-HOXA1 is retrieved by precipitating GST-ERα on glutathione beads, while not with GST alone (N=6, n=1). **(B)** For BiFC assays, COS7 cells were transfected with plasmids encoding human HOXA1 and ERα respectively fused with VC155 and VN173. Unfused VC155 and VN173 were used for negative controls. BiFC signal is observed when VC155-HOXA1 and VN173-ERα are transfected together, while not in negative controls. The insert shows that the BiFC signal provided by the VC155-HOXA1 and VN173-ERα interaction is nuclear (N=4, n=1). Scale bars represent 200 µm; N, number of experiments; n, number of replicates per experiment.

After establishing that HOXA1 and ERα can functionally interact, we next addressed whether HOXA1 and ERα also physically interact. We first performed glutathione co-precipitation (CoP) analysis on cell lysates of HEK293T cells transfected with Flag-hHOXA1 and GST-ERα expression vectors, and monitored fusion protein abundance as a measure of HOXA1-ERα interaction by Western-blotting. Co-expression of unfused GST and Flag-hHOXA1 was used as a negative control. As illustrated in **Figure 5A**, Flag-hHOXA1 was retrieved only by precipitating GST-ERα on glutathione beads. This result shows that HOXA1 protein can bind ERα protein.

In addition, Bimolecular Fluorescence Complementation (BiFC) assays were performed as an independent method to validate HOXA1-ERα protein binding and to establish the intracellular compartment where the interaction occurs. BiFC relies on the complementation between two fragments of the green fluorescent protein Venus (VN173 and VC155). Candidate interactors are fused with VN173 or VC155, and if the proteins of interest interact, VN173 and VC155 reassemble a fluorescent Venus. COS7 cells were transfected with VC155-hHOXA1 and VN173-ERα fusion human proteins. Co-expression of VC155-hHOXA1 with unfused VN173 and reciprocally VN173-ERα with unfused VC155, as well as unfused VN173 and VC155, together were used as negative controls. A three-fold increase in fluorescence signal between test conditions and all three negative controls was applied as a minimal threshold for a valid protein interaction. A significant fluorescence complementation was detected and observed in the nucleus of the cells expressing VC155-HOXA1 and VN173-ERα (**Figure 5B**), thereby confirming HOXA1 and ERα can interact.

### The HOXA1 and ERα Interaction Relies on Various Protein Determinants

**Figure 6:**
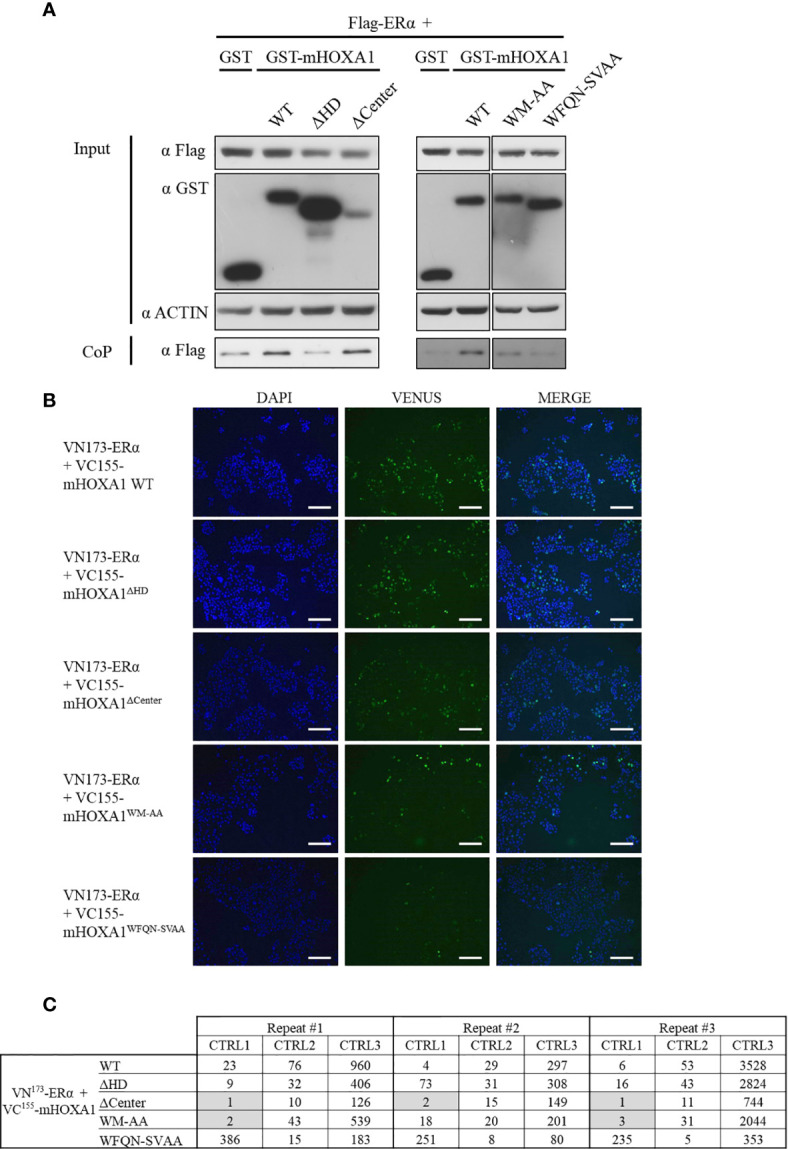
Mapping of HOXA1 regions involved in the HOXA1-ERα interaction. **(A)** For co-precipitation, HEK293T cells were transfected with plasmids encoding Flag-ERα and GST, GST-mHOXA1 WT, GST-mHOXA1^ΔHD^, GST-mHOXA1^ΔCenter^, GST-mHOXA1^WM-AA^ and GST-mHOXA1^WFQN-SVAA^, respectively. GST-mHOXA1 WT interacts with Flag-ERα and the deletion of the central part of HOXA1 does not affect the interaction (N>3). GST-mHOXA1^ΔHD^ loses the interaction with Flag-ERα (N>3). GST-mHOXA1^WM-AA^ and ^WFQN-SVAA^ show weaker interaction with Flag-ERα (N=3/5). **(B)** For BiFC, COS7 cells were transfected with plasmids encoding VN173-ERα and VC155-mHOXA1 WT, VC155-mHOXA1^ΔCenter^, VC155-mHOXA1^ΔHD^, VC155-mHOXA1^WM-AA^ and VC155-mHOXA1^WFQN-SVAA^, respectively. VC155-mHOXA1 interacts with VN173-ERα. VC155-mHOXA1^ΔHD^ interacts with VN173-ERα similarly to the wild type. VC155-mHOXA1^ΔCenter^, VC155-mHOXA1^WM-AA^ and VC155-mHOXA1^WFQN-SVAA^ display weaker BiFC signal than the wild type VC155-mHOXA1 (N=3, n=2). Scale bars represent 200 µm; N, number of experiments; n, number of replicates per experiment. **(C)** BiFC quantification. Fluorescence ratios between the tested condition and the negative control pDest VN173 + VC155-mHOXA1 (CTRL1); the tested condition and the negative control VN173-ERα + pDest VC155 (CTRL2); the tested condition and the negative control pDest VN173 + pDest VC155 (CTRL3). A three-fold increase in fluorescence signal between negative controls and tested conditions was applied as a minimal threshold to conclude for an interaction. Grey boxes indicate where the threshold of >3 is not reached.

To identify the determinants of the molecular interaction between HOXA1 and ERα, mHOXA1 variants were tested in protein CoP and BiFC assays. The interaction between the murine HOXA1 wild type and ERα was first confirmed in CoP of GST-mHOXA1 and Flag-ERα (**Figure 6A**). This interaction was not impaired by the deletion of the central part of HOXA1. However, the variant lacking homeodomain (mHOXA1^ΔHD^) did not show CoP with ERα above background. In addition, the mHOXA1^WM-AA^ and ^-WFQN-SVAA^ mutants showed weaker ERα interaction.

Consistent with CoP assays, BiFC analysis confirmed the interaction between mHOXA1 and ERα (**Figure 6B**). mHOXA1^ΔHD^ showed similar complementation fluorescence signal intensity as HOXA1 wild type, but mHOXA1^ΔCenter^ could no longer bind ERα (**Figures 6B, C**). A decrease in interaction was also observed with both variants with point mutations. The WM to AA mutation negatively impacted the interaction with ERα (**Figures 6B, C**). Within three repetitions, we observed twice loss of interaction with VN173-ERα (**Figure 6B**). Finally, mHOXA1^WFQN-SVAA^ appears slightly impaired in the interaction with ERαHOXA1 wild type (**Figures 6B, C**).

Although the CoP and BiFC assay results are not completely consistent (see **Table 4**), they both show that HOXA1 hexapeptide and homeodomain point mutations decrease HOXA1-ERα interaction.

**Table 4:** Comparative table for CoP and BiFC interaction assays.

Interaction between ERα and mHOXA1 variants	CoP	BiFC
mHOXA1^WT^	+	+
mHOXA1^ΔHD^	–	+
mHOXA1^ΔCenter^	+	+/-
mHOXA1^WM-AA^	+/-	+/-
mHOXA1^WFQN-SVAA^	+/-	+/-

+, positive interaction (by reference to the mHOXA1WT protein); +/-, decreased interaction; -, lost interaction.

**Figure 7:**
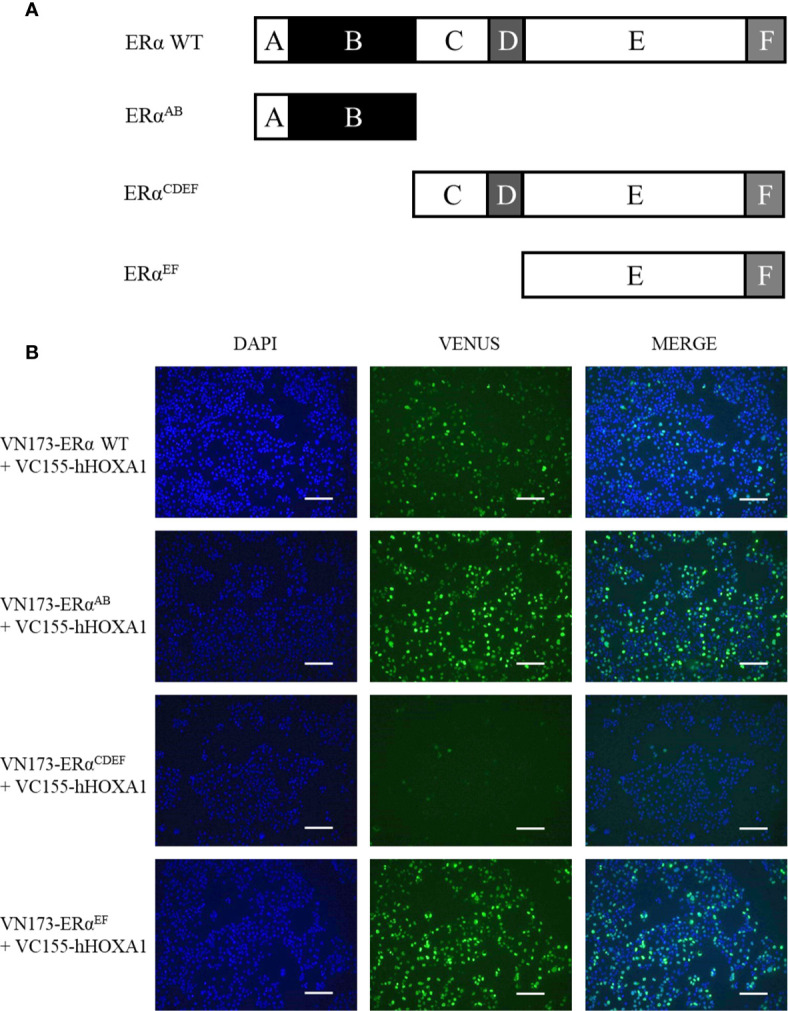
Mapping ERα regions important for the HOXA1 interaction. **(A)** Maps of ERα regions and deletion variants. ERα WT (595 amino acids) is divided in six regions, from A to F In absence of ligand, region A can bind the C-terminal end and represses the transcriptional activity of the protein. Region B spans the transactivation domain AF-1, responsible for co-activator recruitment. Region C is the DNA-binding domain (DBD), structured in two zinc fingers. Region D is a hinge. Region E encompasses the ligand-binding domain (LBD) and the second transactivation function AF-2. Region F is a protein-protein interaction interface. hERα^AB^ is 180 amino acid long, hERα^CDEF^ is 415 amino acid long, hERα^EF^ is 293 amino acid long. **(B)** Interaction between HOXA1 and ERα deletion variants. COS7 cells were transfected with plasmids encoding VC155-HOXA1 and VN173-ERα^AB^, VN173-ERα^CDEF^ and VN173-ERα^EF^ respectively. While ERα^AB^ and ^EF^ variants maintained the interaction with VC155-HOXA1, ERα ^CDEF^ failed, as revealed by the absence of BiFC fluorescence (N≥3, n=2). Scale bars represent 200 µm; N, number of experiments; n, number of replicates per experiment.

Next, ERα variants were analyzed for their ability to bind HOXA1. ERα contains two transactivation domains (called AF) and DNA- and ligand-binding domains (called DBD and LBD, respectively) (8). Along the ERα sequence, six regions can be defined (called A to F, **Figure 7A**). Region A prevents transcription activity in the absence of ERα ligand by binding to the C-terminal end of the protein. Region B contains transactivation domain AF-1. Region C contains two zinc fingers and mediates DNA binding. Region D is a protein hinge. Region E contains a hydrophobic pocket which binds ERα ligands and transactivation domain AF-2. Region F is involved in 14-3-3 protein interactions. BiFC assays (**Figure 7B**) show that only ERα^CDEF^ failed to interact with HOXA1. Both ERα^AB^ and ERα^EF^ showed a slightly increased HOXA1 binding compared to wild type ERα. These data support that CD might impair HOXA1 binding by EF, in the absence of AB.

### HOXA1-Mediated ERα Inhibition Involves Its Ability to Activate the NF-κB Pathway

We previously demonstrated that HOXA1 can activate NF-κB upstream of the IκB inhibitor, probably through the interaction of signaling pathway modulators TRAF2 and RBCK1 (32). In the literature, a large number of positive and negative cross-talks have been identified between the ERα and NF-κB signaling pathways. Many such reports showed that NF-κB and ERα can exert antagonistic activities (54–56). We therefore investigated whether NF-κB could play a role in the HOXA1-ERα antagonism. MCF10A cells were transfected with plasmids encoding Flag-HOXA1 and ERα, together with the *CMV::lacZ* and *ERE::luc* reporter plasmids. Also added was a dominant negative IκBα derivative (IκBα S32/36A, hereafter called IκB-super repressor, or IκB-SR). IκB-SR cannot be phosphorylated by the IKK complex and subsequently degraded. Its interaction with p65/p50 heterodimers inhibits their translocation into the nucleus and impairs NF-κB pathway activity (57, 58).

**Figure 8:**
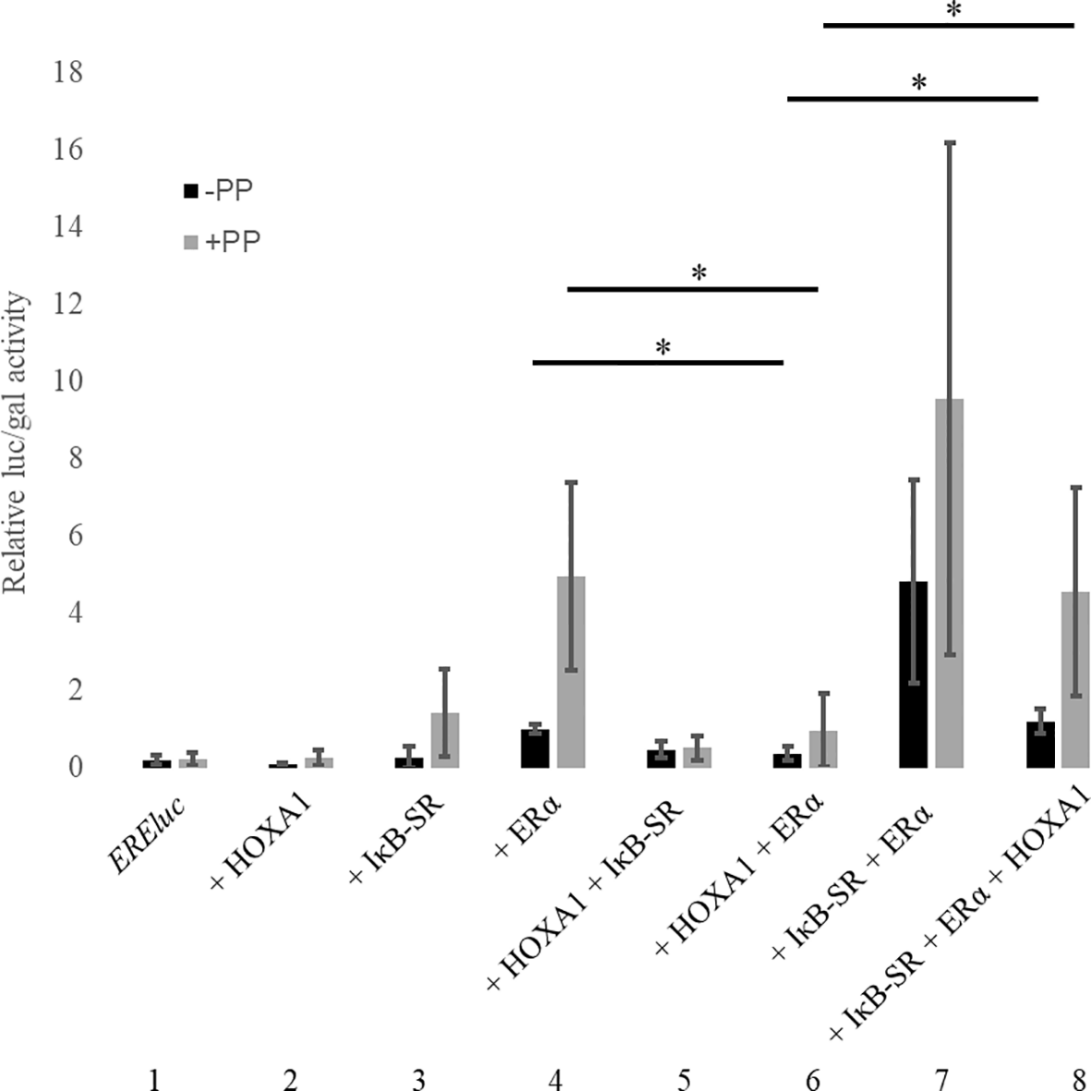
NF-κB pathway involvement in the functional antagonism between HOXA1 and ERα. MCF10A cells were transfected with plasmids encoding *EREluc*, Flag-HOXA1, ERα, IκB-SR, PREP1 and PBX1A and β-galactosidase under the control of a CMV promoter. ER-mediated luciferase activity (luc) was reported to the β-galactosidase activity (gal). See text for details. PP stands for PREP1 and PBX1A. The activation of *EREluc* in presence of ERα was set to 1. * means p < 0,05 (N≥3, n=2). Error bars represent standard deviation for N experiments and n replicates per experiment.

As already observed, HOXA1 impaired ERα activity on its target reporter (**Figure 8**, compare conditions 4 and 6). Addition of IκB-SR had distinct effects on the outcome of the assay. On the one hand, inhibiting NF-κB activity by IκB-SR tends to stimulate ERα (**Figure 8**, compare conditions 4 and 7). On the other hand, IκB-SR significantly diminished the inhibitory effect exerted by HOXA1 on ERα (**Figure 8**, compare conditions 6 and 8). Inversely, HOXA1 was able to antagonize the ERα stimulation provided by IκB-SR. The HOXA1 effects occurred both in the presence and in the absence of the PREP1 and PBX1A, suggesting that HOXA1-mediated inhibition and its release by IκB-SR take place independently of these cofactors known to be critically involved in the HOXA1 transcriptional activity. These data together show that the NF-κB inhibition and HOXA1 expression have opposite effects on ERα, as well as that the NF-κB pathway and HOXA1 functionally interact in inhibiting ERα activity. This supports that the HOXA1-mediated activation of NF-κB is involved in the ERα inhibition.

### ERα Does Not Inhibit HOXA1 Transcription Activity

**Figure 9:**
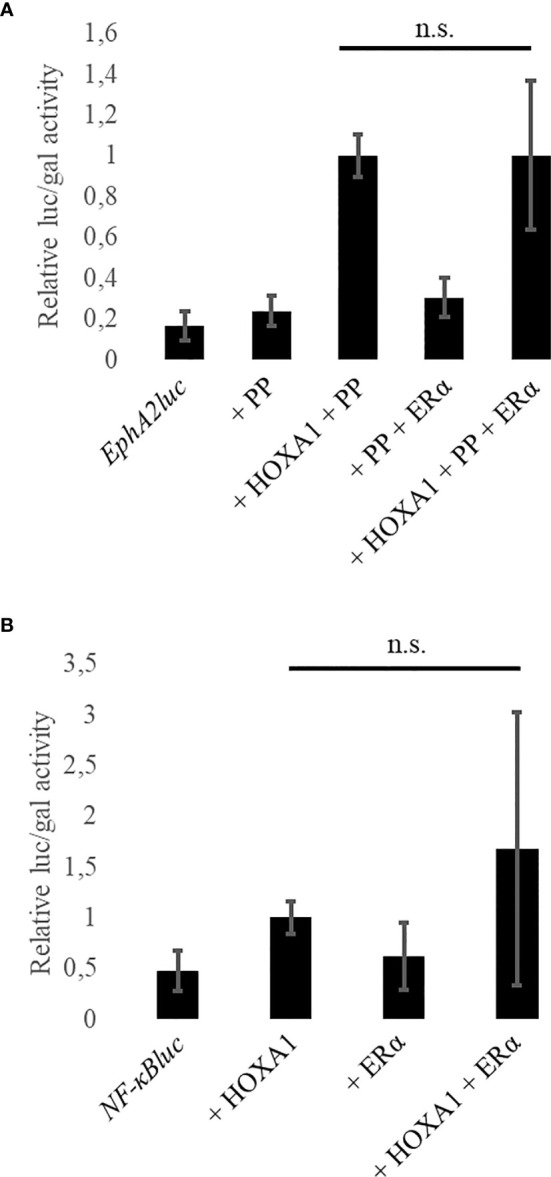
ERα does not interfere with transcriptional and non-transcriptional HOXA1 activities. **(A)** MCF10A cells were transfected with plasmids encoding Flag-HOXA1, PREP1, PBX1A, ERα along with *CMV::lacZ* and *EphA2luc*. Reporter luciferase activity (luc) was reported to the β-galactosidase activity (gal). HOXA1 activates *EphA2luc* in the presence of the cofactors PREP1 and PBX1A (set to 1). The HOXA1-mediated activation is not influenced by the presence of ERα (N=11, n≥2). PP stands for PREP1 and PBX1A. The activation of *EphA2luc* in presence of HOXA1 and PREP1 and PBX1A was set to 1. n.s. : non-significant. **(B)** MCF10A cells were transfected with plasmids encoding Flag-HOXA1, PREP1, PBX1A, ERα along with *CMV::lacZ* and the NF-κB reporter (*NF-κBluc*). NF-κB-mediated luciferase activity (luc) was reported to the β-galactosidase activity (gal). HOXA1 activates the NF-κB reporter (set to 1) and this activation is not influenced by the presence of ERα (N=14, n≥2). n.s., non-significant. Error bars represent standard deviation for N experiments and n replicates per experiment.

Upon demonstrating that HOXA1 can inhibit ERα activity we wanted to test the opposite: the influence of ERα on HOXA1 activation of its known direct target, *EphA2* (30). *EphA2* was shown to be upregulated in TNBC whereas estrogens downregulate it in ER+ breast cancer (59, 60) (reviewed in (61). MCF10A cells were transfected with plasmids encoding Flag-HOXA1, PREP1, and PBX1A, and ERα, together with the *EphA2::luc* reporter plasmid. As expected, HOXA1 together with the TALE cofactors, PREP1 and PBX1A, provided a significant *EphA2::luc* activation (**Figure 9A**). However, this activation was not significantly changed by the presence of ERα. Therefore, in our *in vitro* assays, HOXA1 transcription activity does not appear to be modified by ERα.

### ERα Does Not Inhibit HOXA1-Mediated NF-κB Activation

Finally, we addressed whether ERα could interfere with HOXA1-mediated NF-κB activation. MCF10A cells were transfected with Flag-HOXA1 and ERα expression plasmids, together with an NF-κB reporter plasmid. Again, activity assays revealed that ERα did not significantly inhibit *NF-κB::luc* activation by HOXA1 (**Figure 9B**). Therefore, ERα also does not seem to inhibit the non-transcriptional NF-κB-activation by HOXA1.

## Discussion

Bioinformatic analysis of genome-wide mRNA expression in large public datasets of human breast cancer samples pointed out that *HOXA1* mRNA expression is higher in basal-like breast cancer subtype compared to HER2-enriched, luminal A, and luminal B subtypes. This supports the contribution of HOXA1 in cancer aggressiveness and thereby reinforces its critical role in breast oncogenesis. Bioinformatics also allowed us to reveal an extremely strong, opposite correlation between the HOXA1 mRNA expression and ER status. We identified 2,555 genes whose expression were supportive of a functional antagonism between HOXA1 and ERα. From this starting observation, we confirmed *in vitro* that (1) HOXA1 can inhibit ERα activity. We further demonstrated (2) that this inhibition requires an intact HOXA1 DNA-binding homeodomain and involves its DNA-binding independent capacity to activate NF-κB (32). The HOXA1 action towards ERα is therefore bimodal. We also showed (3) that ERα inhibition does not require HOXA1-PBX interaction. Finally, we provided evidence (4) that HOXA1 and ERα can physically interact in the cell nucleus and that this protein binding relies on various protein determinants but would not be essential for HOXA1 inhibition of ERα transcription activity.

According to our *in vitro* data, HOXA1 can inhibit ERα activity, but ERα cannot repress HOXA1 function. Bioinformatic analysis showed that, among the 2,555 genes shared by the HOXA1 and ERα-associated mRNA expression profiles, about half is upregulated in the presence of HOXA1 but downregulated in ER+ cancers, while the other half shows the opposite correlation. One straightforward scenario would be that HOXA1 can impair ERα activity on both its positively and negatively regulated target genes. *HOXA1* expression has been shown to be strongly correlated to poor prognosis in breast cancer (20). The inhibition HOXA1 exerts on ERα can define one modality of HOXA1 action resulting in cancer aggressiveness. HOXA1 activity would result in conferring gene expression profiles and cell properties similar to ER− cancers. Consistently, low expression levels of microRNAs targeting *HOXA1* are also associated with poor prognosis and Tamoxifen resistance (3, 62–66). Therefore, early *de novo HOXA1* expression in the mammary gland might lead to the development of aggressive subtypes of breast cancer, and late *HOXA1* expression in an ER+ tumor environment might lead to endocrine therapy resistance.

Importantly, Brock et al. demonstrated that *HOXA1* expression repression with siRNAs leads to a decrease in tumor incidence and an increase of ER expressing cells (23). This supports that HOXA1 could interfere with the ER status of mammary cancerous cells and thereby influence the outcome of endocrine therapies. Moreover, in support of such a switch in breast cancer driver, from ERα to HOXA1, Mahajan et al. have shown that *HOXA1* expression in breast cancer can be induced by ERα and that this could be linked to the acquisition of Tamoxifen resistance. Mahajan et al. indeed established that *HOXA1* expression can be triggered by a complex composed of ACK1, ERα, and KDM3A. Upon exposure to heregulin, activation of the receptor tyrosine kinase HER2 results in the phosphorylation and activation of the ACK1 protein kinase. Activated ACK1 then interacts with ERα and phosphorylates the histone demethylase KDM3A. All three partners, ACK1, KDM3A, and ERα, bind to a target site in the first intron of *HOXA1*. KDM3A then removes H3K9 repressive marks and induces *HOXA1* transcription, all in the presence of Tamoxifen (67). The authors finally suggest that the ACK1 activation-HOXA1 expression cascade is involved in ER modulator resistance. In the light of our data, we could therefore propose that after activation by ERα, HOXA1 would exert a negative feedback loop on ERα activity and elicit Tamoxifen resistance.

The HOXA1-mediated inhibition of ERα we highlighted involves the activation of the NF-κB pathway. We have earlier uncovered the ability of HOXA1 to activate the NF-κB pathway after finding strong correlations between the mRNA expression of *HOXA1* and players of the NF-κB signaling network in public datasets of human breast cancer samples (32). Indeed, TNBC was described to have constitutive NF-κB pathway activation (68), which has been associated with endocrine therapy resistance and poor patient outcome (69–71). In complete agreement with this, NF-κB has been shown to be able to repress ERα expression. For instance, (1) NF-κB activates BCL2/RAS signaling and then inhibits *ESR1* expression through the repressor action of the zinc finger protein PRDM1; (2) the serine/threonine kinase PKCθ can both promote NF-κB activity and inhibit *ESR1* expression; (3) NF-κB can activate the methyltransferase EZH2, that can suppress *ESR1* transcription, and is thereby associated with poor outcome to Tamoxifen therapy (70, 72–75). Finally, Oida *et al.* demonstrated that Tamoxifen-resistant MCF7 cells expressed less ERα and that ERα expression can be rescued by inhibiting IKKβ (76). Instead of being involved in repressing expression, our data indicate that NF-κB can inhibit the activity of the ERα protein through its activation by HOXA1. Therefore, HOXA1 and NF-κB could operate a switch in cell growth control by dominating ERα, taking the lead in the oncogenic process and decreasing endocrine therapy sensitivity.

In addition to the functional interaction between HOXA1 and ERα, we also observed that the HOXA1 and ERα proteins can physically bind. This interaction was impaired by the removal of the HOXA1 central part and homeodomain, large protein regions, of 129 and 60 amino acids, respectively. Their deletion could either remove crucial amino acids or disrupt a three-dimensional arrangement involved in protein-protein interaction. Both HOXA1 regions have already been shown to be involved in protein-protein interactions (42, 77). Even more informative are the WM-AA and WFQN-SVAA point mutations, which weakened the interaction with ERα. HOXA1^WM-AA^ is unable to interact with PBX1A and consequently most probably loses its capacity to interact with most or all of its transcriptional targets (46, 52). Mutating WFQN into SVAA removes the conserved glutamine and asparagine of the homeodomain necessary for DNA binding (40). The decreased ERα binding of these HOXA1 mutants, which are also affected in their DNA binding, might indicate that the HOXA1-ERα interaction requires HOXA1 DNA binding. Alternatively, the HOXA1 homeodomain and hexapeptide motif might be directly involved in its docking onto ERα.

All HOXA1 mutants tested in this study are impaired in ERα binding, and three of them also lose the ability to inhibit ERα. HOXA1^WM-AA^ can still inhibit ERα activity despite decreased ERα binding. Therefore, molecular interaction between ERα and HOXA1 might not be necessary to inhibit ERα activity, or slightly impaired binding is not detrimental for HOXA1 functional inhibition of ERα. Alternatively, we cannot exclude that the BiFC and CoP assays might be sensitive enough to detect a loss of molecular interaction, while the luciferase reporter could not be sensitive to a moderate loss of HOXA1-mediated inhibition by the mutant. In any case, the functional significance of the HOXA1-ERα interaction needs further investigation. For example, what needs to be determined is to what extent genes with mRNA expression that is oppositely correlated to HOXA1 and ERα in breast cancer correspond to shared direct target genes, on which the HOXA1-ERα molecular interaction will be translated into a functional transcription output. An important step would be identifying which genes have promoter binding by HOXA1 and/or ERα and determine their regulation. Finally, HOXA1^WFQN-SVAA^ and ^-ΔCenter^ variants can activate NF-κB (32) but cannot inhibit ERα activity (this study). Therefore, a multi-modal mechanism of HOXA1-mediated ERα inhibition might exist for which activation of NF-κB is involved but not sufficient. Another modality of ERα inhibition might involve HOXA1 DNA-binding capacity. A caveat here is that the loss of inhibition capacity revealed by homeodomain deletion or mutation could be not due to their loss of DNA binding, but rather to HOXA1 structure disruption.

HOXA1 is not the only homeodomain protein that functionally interacts with ERα and is correlated with poor prognosis of breast cancer. HOXB13 downregulates ERα expression and activity. Inversely, HOXB7 associates with ER-binding sites to act as an ERα co-activator. HOXB7 binds ERα *via* its homeodomain, in line with the importance of the homeodomain for the HOXA1-ERα interaction. Nonetheless, our data clearly support that unlike HOXB7, HOXA1 inhibits rather than stimulates ERα activity. Remarkably, although their functional interactions with ERα clearly differ, the activities of these three HOX proteins, HOXA1, HOXB7, and HOXB13, have been linked to Tamoxifen resistance and poor prognosis of breast cancer (51, 78–80), most probably reflecting roles in different cancer cell processes, possibly at different stages of tumor development.

## Data Availability Statement

Publicly available datasets were analyzed in this study and referred to, with identifiers, in **Table 3** and **Supplemental Table 1**.

## Author Contributions

MB: Conceptualization, investigation, and writing original draft. BE: Investigation. AT: Investigation. LB: Resources. ND: Resources. DM: Resources. J-FB: Funding acquisition and supervision. DG: Conceptualization, investigation, writing original draft, and writing reviewing and editing. RR: Funding acquisition, supervision, project administration, conceptualization, writing reviewing and editing. All authors contributed to the article and approved the submitted version.

## Funding

MB is a Fonds National de la Recherche Scientifique - FNRS - Télévie grant holder. This work was supported by the Fonds de la Recherche Scientifique -FNRS under Grants n°7.4555.16 and 7.6513.18 and the Fonds Spéciaux de Recherche from UCLouvain.

## Conflict of Interest

The authors declare that the research was conducted in the absence of any commercial or financial relationships that could be construed as a potential conflict of interest.

## Publisher’s Note

All claims expressed in this article are solely those of the authors and do not necessarily represent those of their affiliated organizations, or those of the publisher, the editors and the reviewers. Any product that may be evaluated in this article, or claim that may be made by its manufacturer, is not guaranteed or endorsed by the publisher.

## Footnotes

1 https://www.ncbi.nlm.nih.gov/geo/

2 https://www.ebi.ac.uk/

3 https://portal.gdc.cancer.gov/

4 http://r2.amc.nl

5 http://r2.amc.nl > genome browser

6 http://horfdb.dfci.harvard.edu/hv5/

7 http://vassarstats.net/rsig.html
