# Supplementary material for: HOXA1 Is an Antagonist of ERα in Breast Cancer
Source: Front Oncol. 2021 Aug 18;11:609521. doi: 10.3389/fonc.2021.609521 (PMC8417444; doi:10.3389/fonc.2021.609521)
Supplement: Supplementary file 1 [file DataSheet_1.pdf]

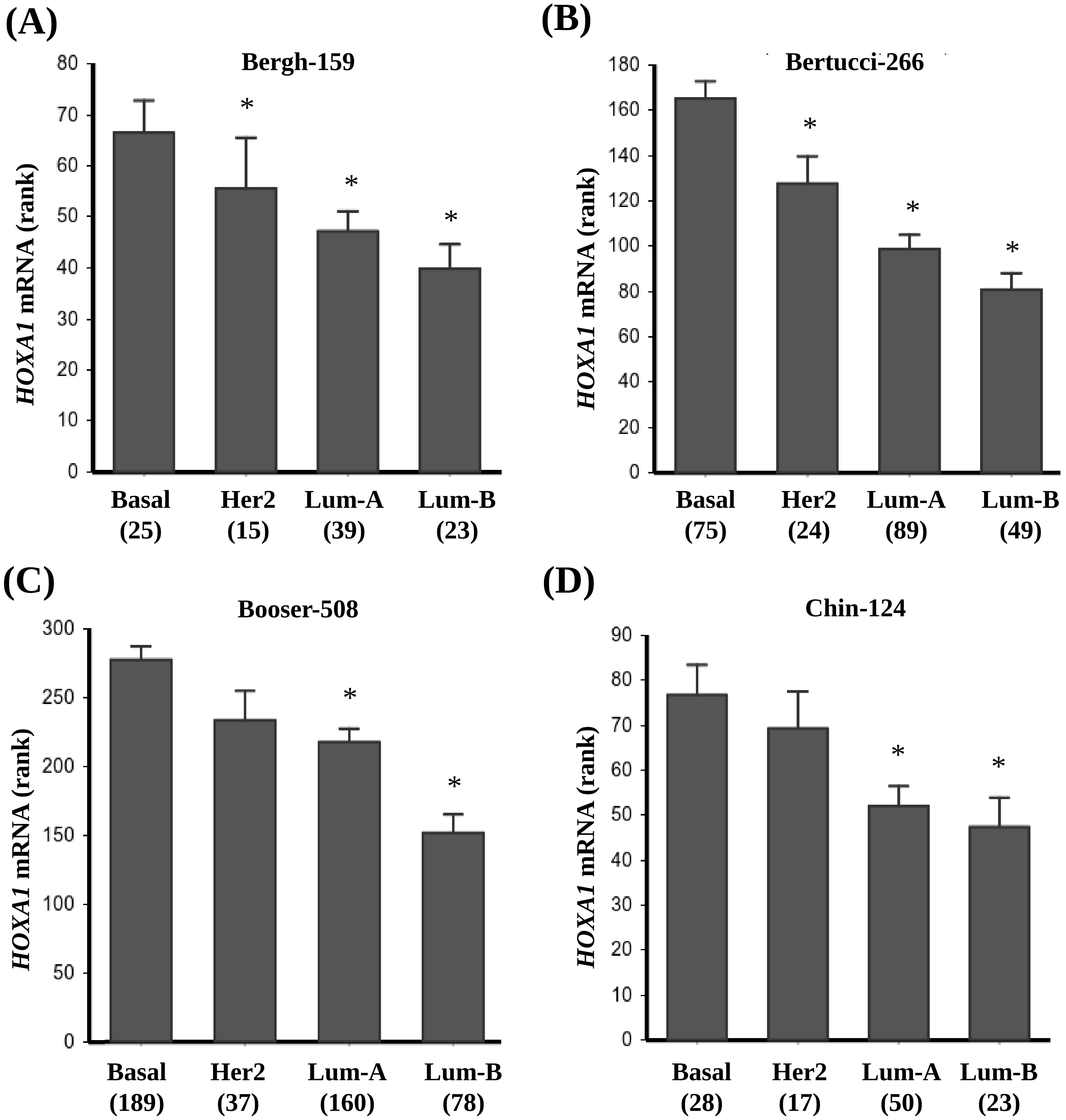

Supplemental Figure 1. High *HOXA1* mRNA expression correlates with the basal breast cancer subtype. *HOXA1* mRNA expression correlation with breast cancer molecular subtypes. Panels A-D represent the results from all 4 breast cancer datasets in the public domain with sample number > 100, and annotation on molecular subtype: Bergh-159, Bertucci-266, Booser-508, and Chin-124, respectively. Below the graphs are the different subtypes: basal-like (basal), HER2-overexpressing (Her2), Luminal-A (Lum-A), and Luminal-B (Lum-B), between brackets are the number of samples per subtype. Vertical bars represent the standard error of the mean. * denotes significant difference with the basal subtype expression ($p < 0.05$).

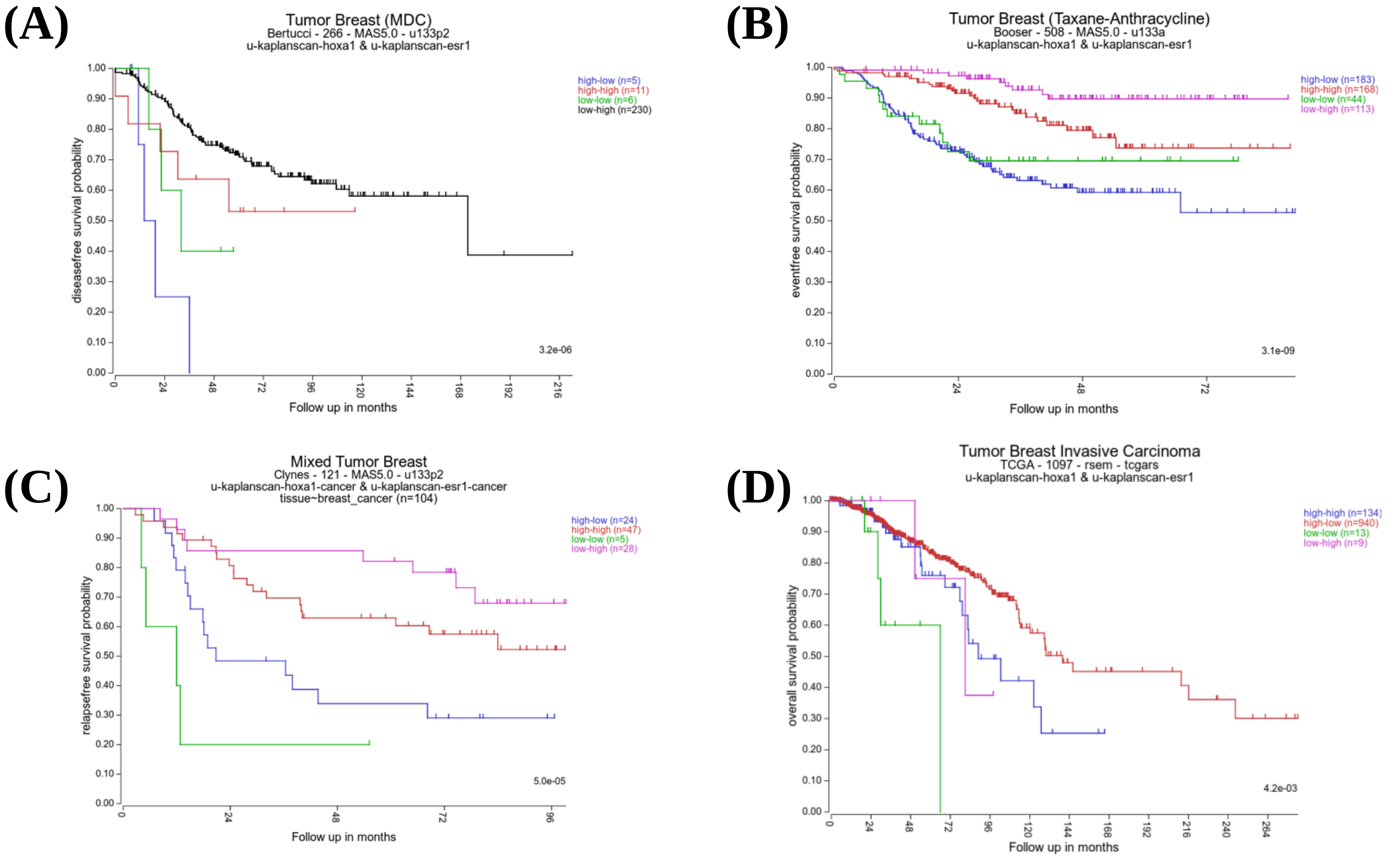

Supplemental Figure 2. Kaplan-Meier curves for the datasets for which survival data was available: Bertucci-266 (A), Booser-508 (B), Clynes-121 (C) and TCGA-1097 (D). Each vertical step in the curve indicates one or more events (e.g. death, relapse). Right-censored patients are indicated by a vertical mark in the curve at the censoring time. Visual inspection suggests that survival seems to be more favorable for patients with lower levels of HOXA1 and higher levels of ESR1 in A, B and C. The log-rank test indicates a significant difference between the survival curves.
